# MZB1 regulates the immune microenvironment and inhibits ovarian cancer cell migration

**DOI:** 10.1515/med-2025-1174

**Published:** 2025-05-13

**Authors:** Mingyue Zhu, Guxin Zhou, Fangyuan Chang, Jin Liu

**Affiliations:** Department of Obstetrics and Gynecology, The Affiliated Hospital of Jiangsu Vocational College of Medicine, KANGFU West Road, Dongtai City, Jiangsu, 224200, China

**Keywords:** ovarian cancer, MZB1, tumor immune microenvironment, immune infiltrate cell, nomogram

## Abstract

**Background:**

The immune microenvironment of ovarian cancer is crucial in its progression. Recent studies highlight the significant role of MZB1 in shaping the tumor immune microenvironment (TIME), although its specific function in ovarian cancer remains unclear.

**Materials and methods:**

We analyzed 381 ovarian cancer samples from the TCGA database, along with additional samples from GEO and single-cell datasets. Differentially expressed genes (DEGs) were identified using DESeq2. Various algorithms were applied to assess the relationship between MZB1 and the TIME. Cell proliferation was measured using the CCK-8 assay, while cell migration was evaluated using the wound healing assay. Furthermore, a nomogram predicting overall survival was developed based on multivariable Cox regression results.

**Results:**

Our results indicated a positive correlation between high MZB1 expression and improved clinical prognosis. Additionally, higher MZB1 expression was linked to increased immune cell infiltration within the TIME. Elevated MZB1 levels inhibited the migration and proliferation of SKOV3 cells. The nomogram’s C-index was 0.702, and its calibration curve demonstrated good calibration, indicating satisfactory discrimination and accuracy in predicting patient outcomes.

**Conclusions:**

This study comprehensively analyzes MZB1’s role in the TIME of ovarian cancer. MZB1 is a promising prognostic marker and a potential target for personalized treatment.

## Graphical abstract

**Figure:**
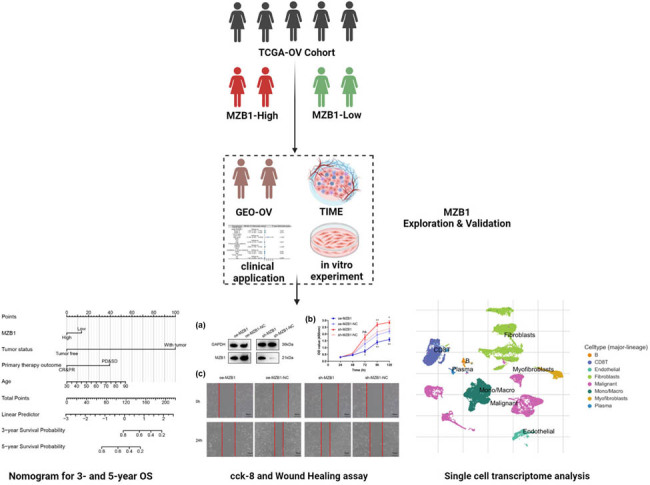

## 1 Introduction

Ovarian cancer is the deadliest malignancy among women, often diagnosed at an advanced stage with a poor prognosis [1]. Characterized by its elusive nature and early metastasis [2,3], the 5-year survival rate remains around 40% [4]. The high recurrence rate of 82% among advanced-stage ovarian cancer patients after treatment significantly affects their quality of life and imposes a substantial burden on societal resources [4,5,6].

Ovarian cancer treatments, including surgery, radiotherapy, and chemotherapy, are complemented by emerging therapies like PARP inhibitors, which extend survival in advanced-stage patients with BRCA1/2 mutations, and PD-1/PD-L1 inhibitors, which enhance immune responses against tumors [7,8,9,10,11,12]. Targeted therapies have proven to be effective in treating ovarian cancer. Therefore, the identification of novel clinical biomarkers could be instrumental in advancing our understanding of ovarian cancer’s diagnosis, prognosis, and treatment strategies.

Marginal zone B and B-1 cell-specific protein 1 (MZB1) is localized in the endoplasmic reticulum, playing critical roles in B-cell calcium homeostasis, cell motility, and integrin-mediated T-cell adhesion [13,14]. MZB1 is an immune-related protein, and its upregulation is associated with autoimmune diseases such as periodontitis, systemic lupus erythematosus, and multiple sclerosis [13]. Research on MZB1 in tumors has been relatively scarce, but it has recently been increasingly recognized. Wu et al. demonstrated that MZB1 acts as a tumor suppressor and is downregulated in colorectal cancer tumor tissues [15]. In primary gastric cancer, low MZB1 expression is correlated with a higher risk of recurrence following curative resection, as MZB1’s suppressive role links its reduced levels to increased hematogenous recurrence and changes in tumor progression-related gene expression [16]. Notably, as MZB1 was initially identified in immune cells, its role in the regulation of the TIME warrants close attention. While research on MZB1 in cancer-related diseases holds significant promise, regrettably, its role in ovarian cancer remains unexplored. We hypothesize that MZB1 may be a critical target influencing the progression of ovarian cancer. Therefore, further studies are urgently needed to elucidate the function and underlying mechanisms of MZB1 in this context.

In this study, we leveraged the TCGA, GEO, and other databases to comprehensively analyze MZB1, including its expression patterns, associations with survival outcomes, genetic alterations, immune infiltration, and involvement in cellular pathways. Furthermore, CCK8 and wound healing assays were performed to evaluate the impact of MZB1 on cellular phenotypes, providing insights into its functional roles and potential mechanisms in the pathogenesis and clinical prognosis of ovarian cancer. This comprehensive analysis aims to uncover MZB1’s potential as a biomarker and therapeutic target, paving the way for improved personalized treatment strategies.

## 2 Materials and methods

### 2.1 Data collection and pre-processing

We obtained RNA sequencing data and corresponding clinical follow-up information for 381 samples from the TCGA database (excluded if clinicopathological information is missing). The RNAseq data, initially in FPKM format, were converted to TPM format and log2-transformed. Duplicate samples were excluded to retain relevant clinical information. Two GEO database platforms (GPL570 and GPL96) were used to validate the MZB1 expression.

### 2.2 Protein–protein interaction (PPI) networks

To understand MZB1’s functions and efficacy, we constructed a PPI network using the STRING database (https://cn.string-db.org). We set a minimum interaction score of 0.7, with interaction predictions based on experimental evidence and curated databases, allowing visualization and exploration of MZB1’s interactions and potential roles.

### 2.3 Differential expression and survival analysis of MZB1

We obtained RNA-seq data for ovarian cancer patients from the TCGA database and normal ovarian tissue samples from the GTEx project via UCSC Xena. Kaplan–Meier survival analysis was performed to evaluate the correlation between MZB1 expression levels and patient prognosis. The log-rank test was used to analyze the differences in OS, PFI, and DSS.

### 2.4 Immune infiltration analysis

Immune cell infiltration and gene expression regulation were analyzed using TIMER, quantifying six immune cell types per sample. CIBERSORT was used to calculate relative scores for 24 immune cell types, predicting immunocyte phenotypes. The correlation between MZB1 and immune cell infiltration was assessed using the xCell algorithm.

### 2.5 Gene Set Enrichment Analysis (GSEA)

GSEA was conducted employing the “clusterProfiler” R package to explore biological signaling pathways in the high- and low-expression groups of MZB1. The top five pathways were identified and presented. The enrichment results were based on the net enrichment score (NES), false discovery rate (FDR), and *p*-value. Gene sets meeting the criteria of |NES| > 1, adjusted *p*-value < 0.05, and FDR *q*-value < 0.25 were considered statistically significant.

### 2.6 Single-cell transcriptome analysis

We employed the publicly available online database Tumor Immune Single-cell Hub 2 (TISCH2, http://tisch.comp-genomics.org/home/) for the single-cell transcriptome analysis of pre-existing data. TISCH2 provides comprehensive cell-type annotation at the single-cell level, facilitating the investigation of the TIME in various cancer types.

### 2.7 Cell culture

Ovarian cancer SKOV3 cells were cultured in DMEM supplemented with 10% fetal bovine serum (FBS), 100 IU/mL penicillin, and 10 µg/mL streptomycin at 37°C in a humidified atmosphere with 5% CO_2_.

### 2.8 Cell transfection

MZB1 shRNA targeting sequence: shMZB1: 5′-GGACUACGGAGUUCGAGAATT-3′. SKOV3 cells were transfected with MZB1 shRNA or scrambled shRNA plasmids using ExFect Transfection Reagent (Vazyme) at 37°C for 24 h. Human MZB1 cDNA (XM_054352720) was amplified and inserted into pLV6ltr-ZsGreen-Puro-CMV, followed by transfection using ExFect Transfection Reagent.

### 2.9 Cell proliferation assay (CCK-8)

Transfected SKOV3 cells were seeded into 96-well plates (1 × 10^4^ cells/well) and incubated at 37°C, 5% CO_2_. At 0, 24, 48, 72, 96, and 120 h, the medium was replaced with 90 μl of fresh medium and 10 μl of CCK-8 (APExBIO; Cat. No. K1018) per well. After incubation at 37°C, the OD values at 450 nm were measured using a microplate reader to evaluate cell proliferation.

### 2.10 Migration assay

For the wound healing assay, 5 × 10^5^ cells were plated in 6-well plates. A scratch was made using a 20 μm pipette tip, and cells were washed with DMEM and incubated at 37°C for 24 h. Images were taken at 0 and 24 h to document wound closure.

### 2.11 Western blot

Cells were homogenized and sonicated in ice-cold RIPA buffer. Proteins were extracted, denatured, and separated on a 12.5% SDS-PAGE gel. Membranes were incubated overnight at 4°C with primary antibodies (MZB1, Proteintech; 1:1,000), followed by 1 h at room temperature with secondary antibodies (Goat Anti-Rat IgG; Biosharp; 1:10,000). Chemiluminescence was used for detection.

### 2.12 Construction and verification of nomogram

Variables with *p*-values ≤ 0.1 in univariate analysis were included in multivariate Cox regression. Independent prognostic factors (*p* < 0.05) were used to construct nomograms. The C-index assessed discriminative performance, and calibration curves evaluated nomogram accuracy.

### 2.13 Statistics

Statistical analyses and graphical presentations were conducted utilizing R software (version 4.1) equipped with the “ggplot2” and “ggpubr” packages. The false discovery rate (FDR) method was applied to adjust *p* values. For categorical data, the chi-square test was employed, whereas Wilcoxon’s rank-sum test and Student’s *t*-test were utilized for assessing continuous variables. Correlation analysis was performed using the Spearman method. Significance levels were indicated as follows: ****p < 0.0001, ***p < 0.001, **p < 0.01, *p < 0.05, and ns for non-significant results.


**Ethics approval and informed consent:** Not applicable.

## 3 Results

### 3.1 Elevated expression of MZB1 is associated with a favorable prognosis

**Figure 1:**
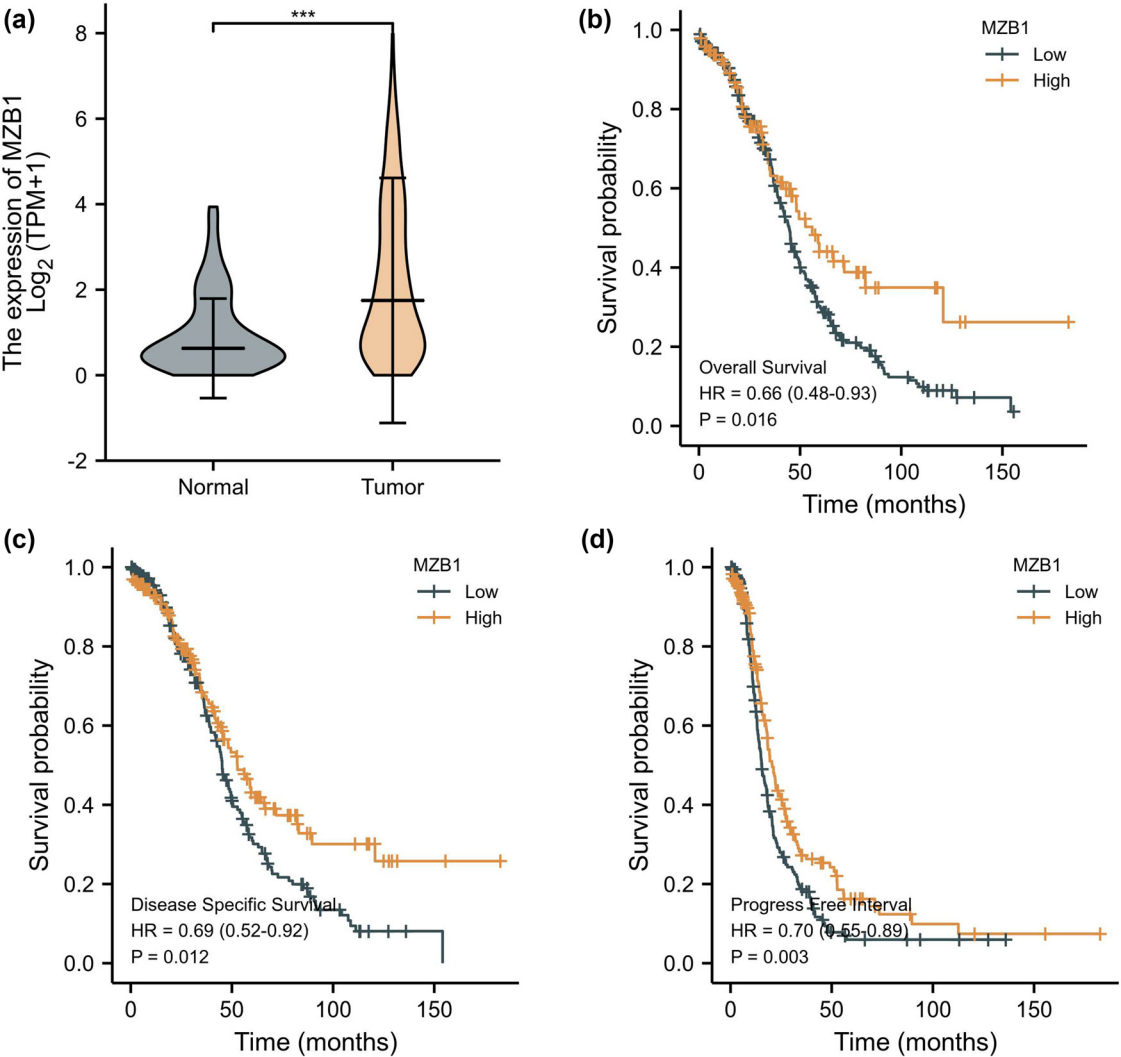
MZB1 expression levels in ovarian cancer and its impact on prognosis: (a) expression of MZB1 in tumor tissues and corresponding normal tissues; and (b)–(d) Kaplan–Meier curves show that lower expression of MZB1 is associated with poorer prognosis. ***p < 0.001.

**Figure 2:**
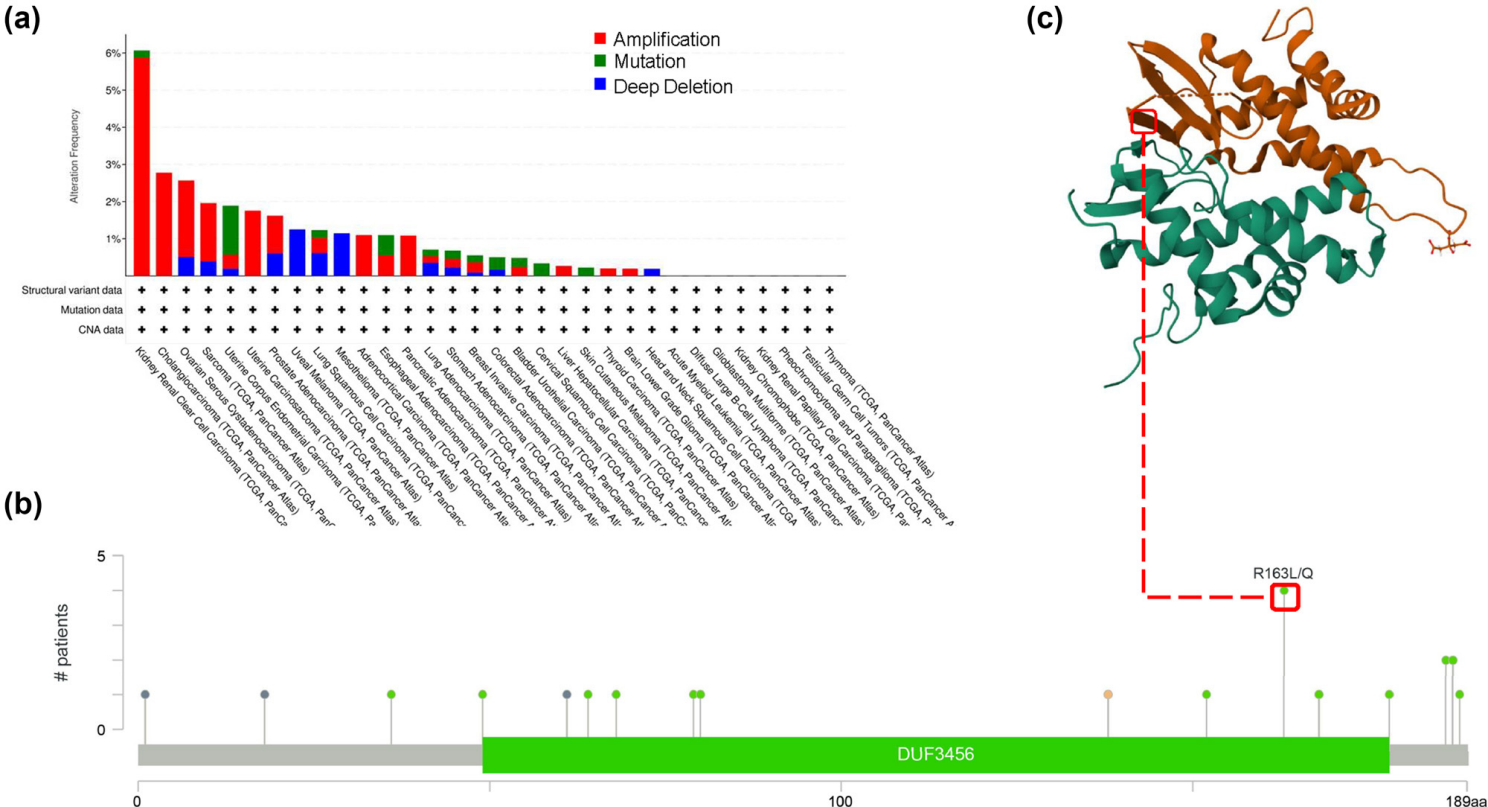
Mutation features of MZB1 in pan-cancer: (a) alteration frequency with mutation type of MZB1; (b) alteration mutation site of MZB1 genetic alteration; and (c) the mutation site with the highest alteration frequency (R163L/Q) in the 3D structure of MZB1.

MZB1 expression was upregulated in ovarian cancer tissues compared to normal ovarian tissues (Figure 1a; Figure S1a and b). High expression was associated with better OS (Figure 1b), and patients with high expression had longer disease-specific survival (DSS) and progression-free interval (PFI) compared to those with low expression (Figure 1c and d). This suggests that high MZB1 expression is linked to a favorable prognosis in patients, indicating its potential as a prognostic marker. In TCGA tumor samples, ovarian cancer had the highest frequency of MZB1 genetic alterations, primarily amplifications (Figure 2a). Figure 2b shows the sites of MZB1 genetic alterations, including the R163L/Q alteration (Figure 2c). However, ovarian cancer cases with MZB1 alterations showed no OS advantage compared to those without alterations (log rank *p* = 0.964, Figure S1c). These findings suggest that higher MZB1 expression predicts better patient prognosis, while MZB1 gene alterations do not affect its role in ovarian cancer progression.

### 3.2 MZB1 is involved in DNA damage response and cell cycle regulation

**Figure 3:**
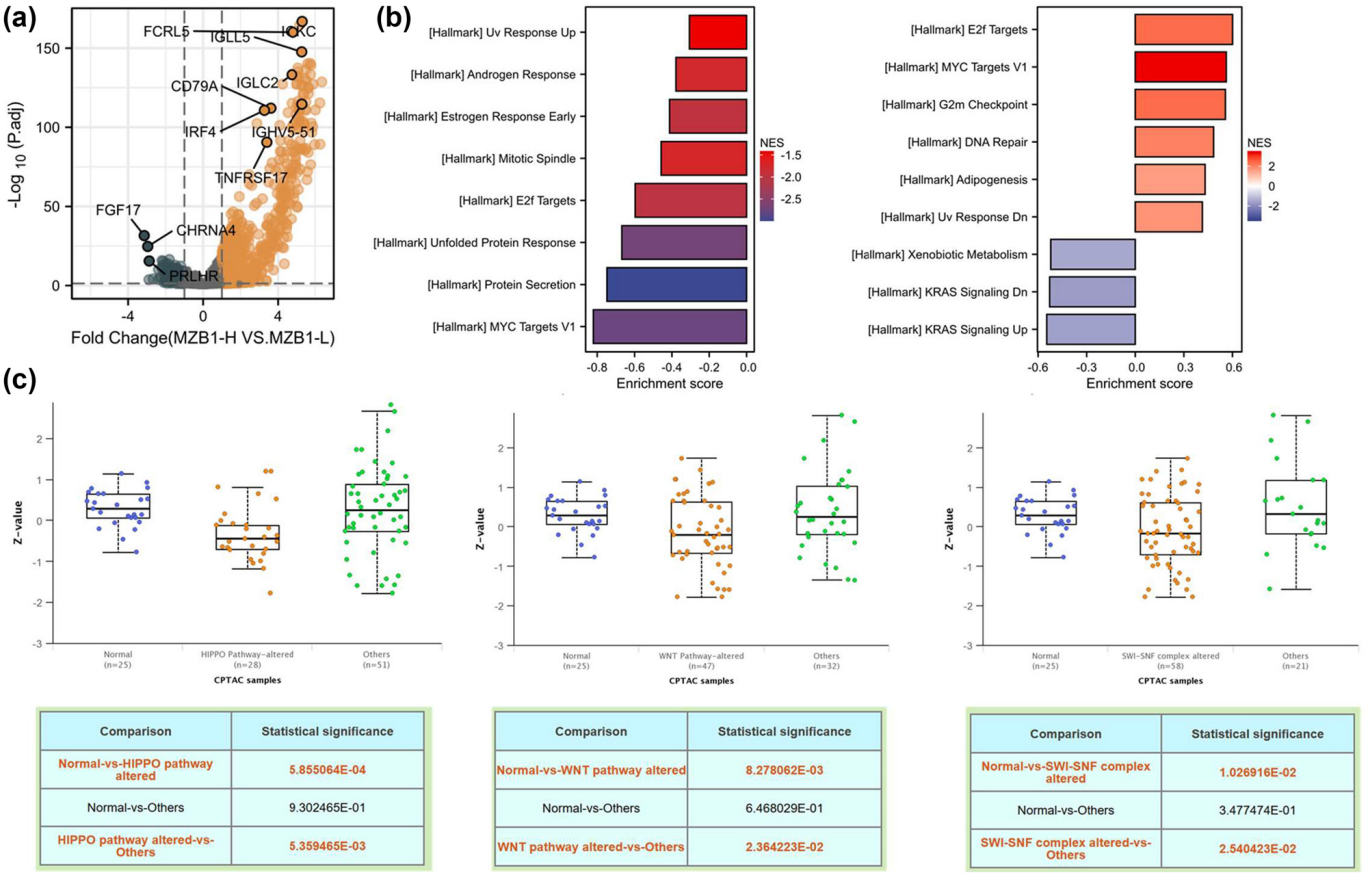
MZB1 is involved in multiple signaling pathway responses: (a) differentially expressed genes shown by volcano plot; (b) GSEA pathways related to DEGs; and (c) impact of pathway mutations on MZB1 expression.

Using DEseq2, we identified 3,510 upregulated and 5,173 downregulated genes associated with MZB1 expression (Figure 3a). GSEA revealed that these genes are involved in DNA damage response and cell cycle regulation pathways (Figure 3b) and potentially in steroid hormone response, HIPPO, and WNT pathways (Figure 3c). These pathways are associated with immunity and tumor proliferation, suggesting that MZB1 may regulate tumor progression by modulating immune responses and tumor proliferation. Additionally, the PPI network indicated that MZB1 interacts mainly with proteins related to the cell cycle and endoplasmic reticulum stress (Figure S1d). Therefore, differences in endoplasmic reticulum stress-related proteins were analyzed under different MZB1 expression states (Figure S2a). The relationship between MZB1 and endoplasmic reticulum stress has not been reported, warranting further experimental investigation. These findings highlight MZB1’s role in targeted and immunotherapy strategies. This also provides a direction for future in-depth mechanistic studies.

### 3.3 MZB1 has been detected in ovarian cancer cells

**Figure 4:**
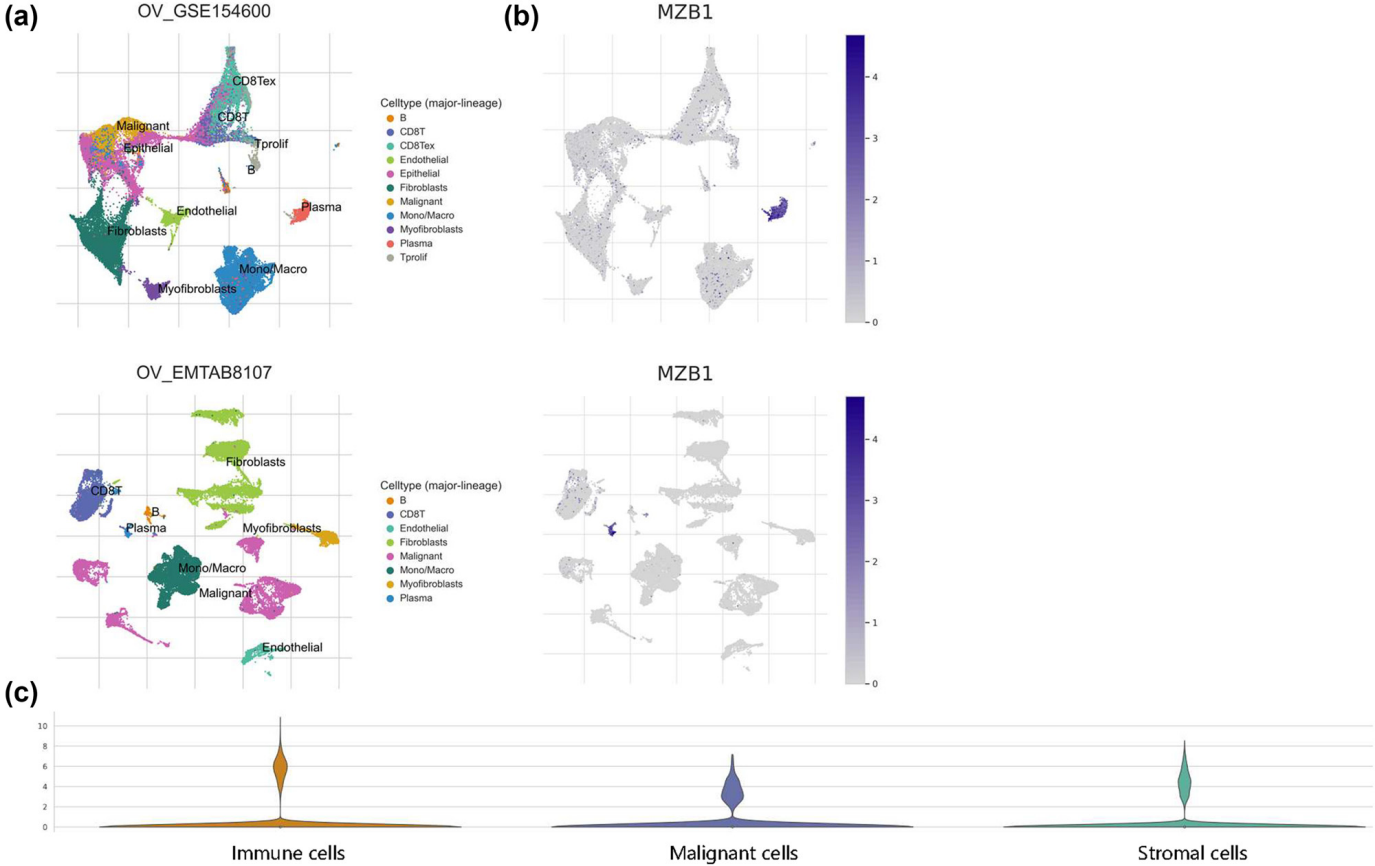
Expression of MZB1 in tumor cells and immune cells: (a) single-cell annotation plot; (b) single-cell expression of MZB1 in OV cohorts; and (c) grid violin plot detailing the average expression distribution of MZB1 on each type of cell (tumor, immune, and stromal cells).

Single-cell analysis of the ovarian cancer dataset GSE154600 showed MZB1 expression in both ovarian cancer cells and immune cells, predominantly plasma cells, with relatively lower expression in tumor cells (Figure 4a–c). This explains the high MZB1 expression observed in bulk RNA sequencing of ovarian cancer tissues. MZB1 in ovarian cancer cells may directly impact tumor cells, while MZB1 in immune cells may indirectly influence tumor progression by regulating the function of the respective cells. Further analysis in multiple ovarian cancer single-cell databases confirmed these findings (Figure S2b). MZB1 exhibited broader expression in ovarian clear cell carcinoma compared to other ovarian cancer types (Figure S2c; Table S1), highlighting the importance of further investigating its role in this particular subtype of ovarian cancer. The results suggest that MZB1 may regulate ovarian cancer progression through multiple mechanisms by influencing both ovarian cancer cells and immune cells in the TIME.

### 3.4 MZB1 plays a crucial role in the TIME of ovarian cancer

**Figure 5:**
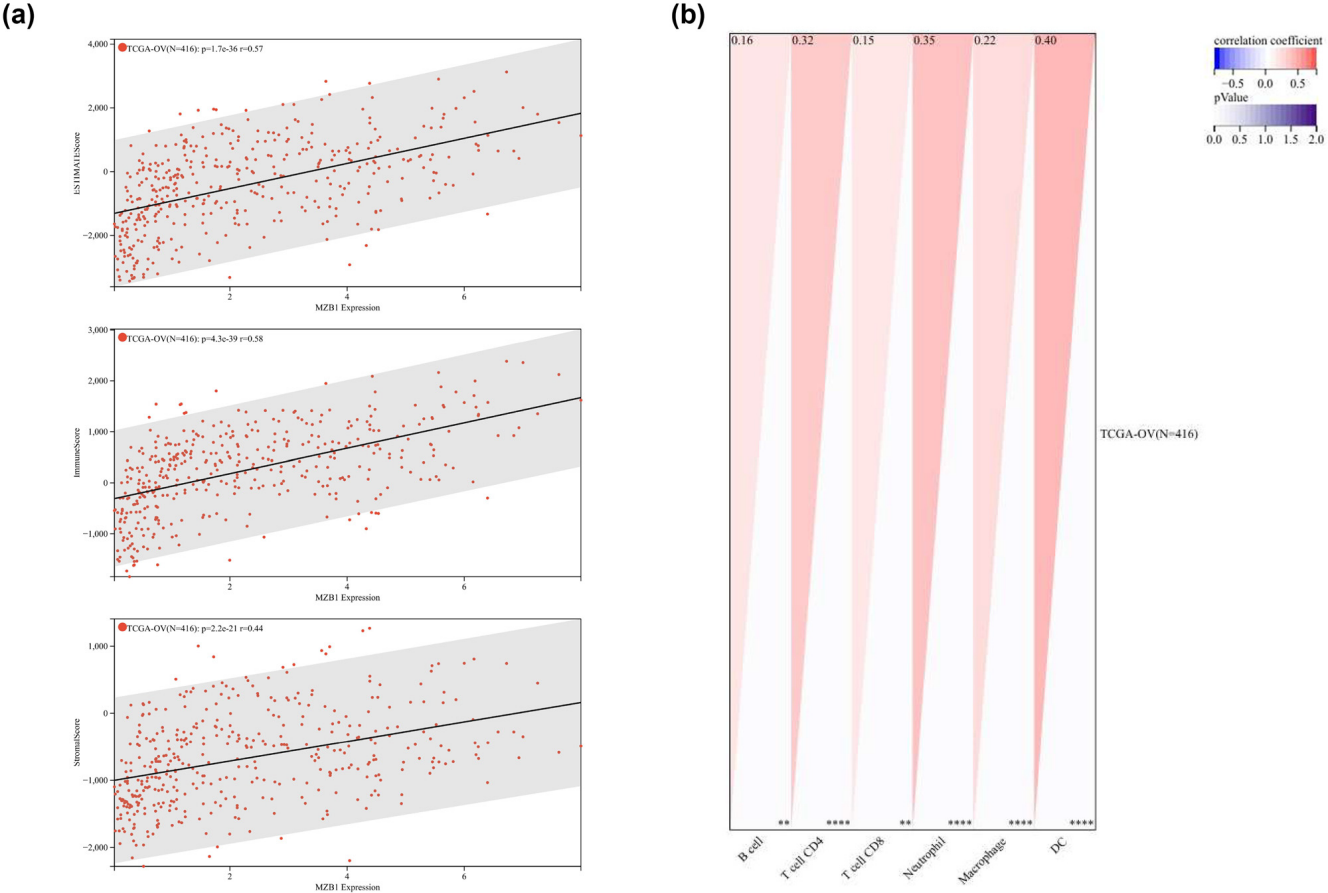
MZB1 is closely associated with the immune microenvironment. Estimate (a) and Timer (b) algorithms were used to calculate the correlation between MZB1 expression and immune cells. *Spearman*; **p < 0.01; ****p < 0.0001.

Using ESTIMATE, TIMER, XCELL, and CIBERSORT algorithms, we investigated the correlation between MZB1 and various immune cells in the ovarian cancer microenvironment (Figure 5a and b; Figure S3a and b). These cells play a crucial role in the tumor microenvironment and are an essential component of the TIME. MZB1 showed significant positive correlations with B cells, T cells, and dendritic cells (Figure S3c and d), which often exert anti-tumor effects. High MZB1 expression correlated with increased immune cell infiltration and higher immune scores, suggesting MZB1’s positive regulatory role in the tumor immune response, explaining the better prognosis for patients with high MZB1 expression.

### 3.5 MZB1 inhibits the migration of SKOV3 by affecting its proliferation

**Figure 6:**
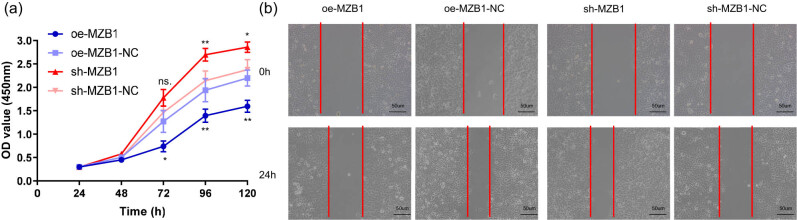
MZB1 inhibits the migration and proliferation of SKOV3 cells: (a) proliferative ability of SKOV3 assessed by CCK-8 assay; and (b) wound-healing assays were conducted at 0 and 24 h in SKOV3 cells with MZB1 overexpression or knockdown. *N* = 3; *p < 0.05; **p < 0.01; ns.: no significance.

To investigate the role of MZB1 in ovarian cancer cells, we conducted cellular assays. CCK-8 assay showed that MZB1 significantly affected SKOV3 cell proliferation (Figure 6a). Overexpression of MZB1 inhibited cell proliferation, while knockdown of MZB1 enhanced proliferative capacity. Wound healing assays demonstrated that MZB1 knockdown enhanced SKOV3 cell migration, while overexpression inhibited migration (Figure 6b). The results indicate that MZB1 within tumor cells plays a role in suppressing tumor proliferation and migration, functioning as a tumor suppressor gene.

### 3.6 miR-1193 is a potential upstream regulatory molecule

Using TargetScan and mirDIP prediction tools, we identified miR-1193 as a potential upstream regulator of MZB1 (Figure S4a and b). miR-1193 is implicated in various biological processes and may modulate MZB1 expression. Further studies are needed to elucidate the molecular mechanisms underlying miR-1193’s regulation of MZB1 and its role in cancer pathways.

### 3.7 Exploration of the clinical application value of MZB1

**Figure 7:**
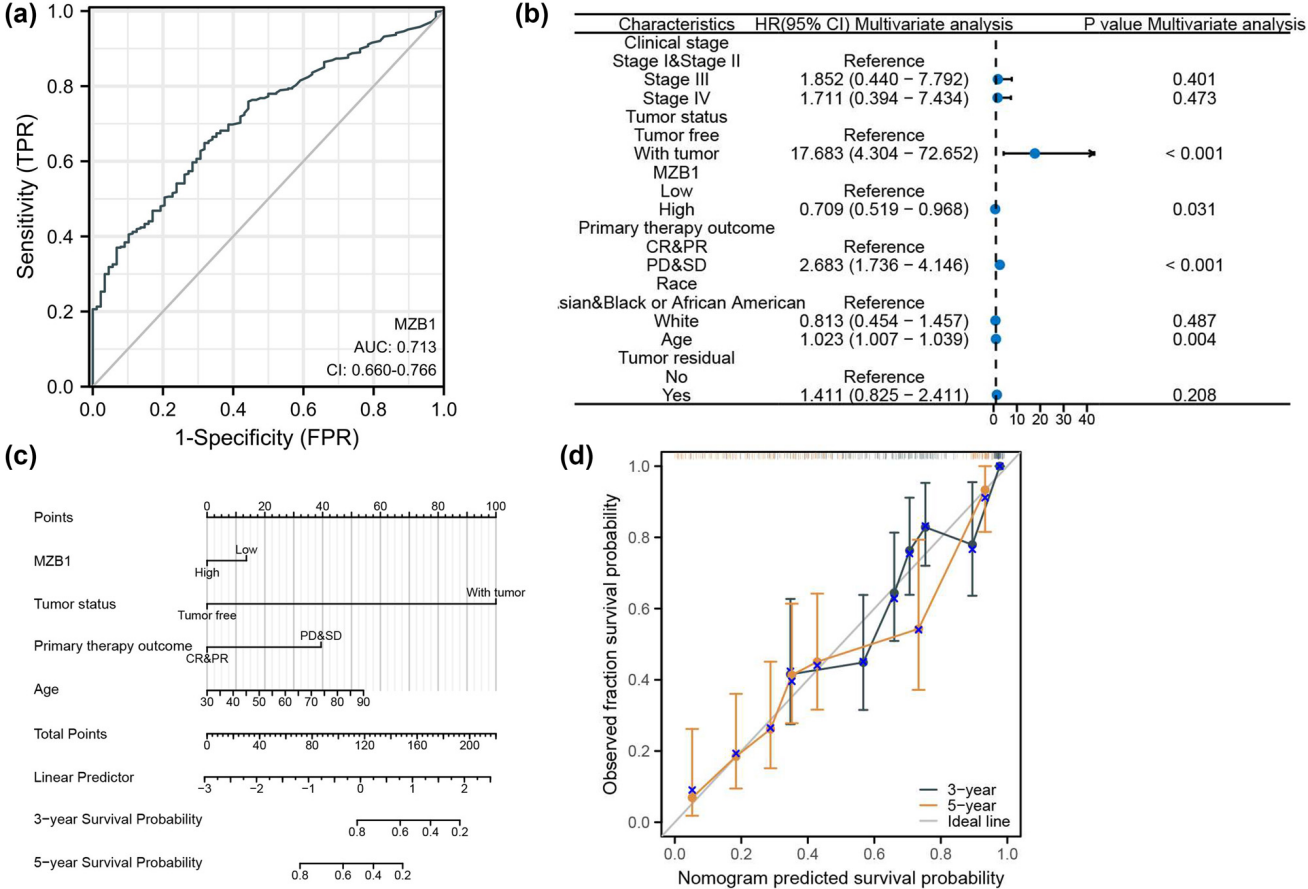
The clinical value of MZB1: (a) diagnostic efficacy of MZB1 in ovarian cancer; (b) Forest plot showing MZB1 as an independent prognostic factor; (c) establishment of OS nomogram; and (d) calibration curve for nomogram predictions.

We explored MZB1’s clinical potential and found it valuable (AUC > 0.70) for diagnosing ovarian cancer (Figure 7a). Cox regression analysis identified MZB1 as an independent prognostic factor (Figure 7b). A prognostic nomogram based on multivariable Cox regression analysis demonstrated good discriminatory ability (C-index: 0.702, 0.680–0.724) and satisfactory calibration (Figure 7c and d). This indicates that we have preliminarily developed a prognostic prediction model with clinical application value. MZB1 holds significant clinical application potential in ovarian cancer.

## 4 Discussion

The present study establishes MZB1 as a novel prognostic biomarker and therapeutic target in ovarian cancer through multi-omics analyses and experimental validation. Our findings reveal three key aspects of MZB1’s involvement in ovarian carcinogenesis: (1) its tumor-suppressive functions through direct modulation of cancer cell proliferation/migration, (2) its critical immunomodulatory role within the TIME, and (3) its unique expression patterns in chemoresistant histological subtypes, particularly ovarian clear cell carcinoma (OCCC). These insights significantly extend the current understanding of MZB1’s multifaceted roles beyond its established functions in autoimmune diseases and other malignancies [13,15,16].

The clinical potential of MZB1 deserves substantial attention, particularly in early disease diagnosis and prognostic prediction, areas that remain significantly underexplored in current research [17,18,19]. Our multi-omics approach demonstrated that elevated MZB1 expression correlates with improved OS, DSS, and PFI. This prognostic value remained significant after adjusting for clinicopathological variables in multivariate Cox regression, indicating MZB1’s potential as an independent prognostic marker. The functional basis for this observation was elucidated through *in vitro* experiments showing that MZB1 knockdown enhances SKOV3 cell proliferation and migration, while overexpression exerts the opposite effects. These results collectively position MZB1 as a tumor suppressor in ovarian cancer. High MZB1 expression is an independent favorable prognostic factor, also attributable to its positive regulation within the ovarian cancer immune microenvironment [20,21]. Therefore, it is crucial to investigate the features of TIME, immune status, and relevant immunotherapy approaches to better understand the regulatory mechanisms of the immune microenvironment, enabling precise immune intervention in tumors [22,23].

The TIME dynamically regulates MZB1 activity, which in turn modulates cancer cell behavior [24,25]. Interestingly, the observed upregulation of MZB1 in various tumors presents an apparent paradox, given its documented tumor-suppressive properties. This phenomenon may be resolved by considering MZB1’s dual functionality in immune regulation – while it directly suppresses malignant cell migration through cancer cell-intrinsic mechanisms [26], it simultaneously engages in immunomodulatory processes within the TIME. Our single-cell analysis revealed MZB1 expression in both tumor cells and immune subsets, while bulk RNA-seq correlations with immune infiltration scores suggest dual roles in cancer cell biology and immune modulation. Elevated MZB1 expression in plasma cells, T cells, and other immune cells in the TIME contributes to anti-tumor effects and inhibits tumor progression [27,28]. Specifically, MZB1 showed strong positive associations with anti-tumor immune populations, including CD8^+^ T cells (*p* < 0 .001) and dendritic cells (*p* < 0.001). The elevated immune scores in MZB1-high tumors further support its role in fostering an immunocompetent TIME, which may synergize with checkpoint inhibitors – a hypothesis requiring validation in immunotherapy trials. Moreover, MZB1 may exert an anti-tumor effect by activating the complement system, which aligns with its role in various tumors [29].

A particularly intriguing finding was the elevated MZB1 expression in OCCC, a subtype characterized by platinum resistance and poor outcomes [30]. OCCC exhibits intrinsic resistance to platinum-based therapy, with chemotherapy response rates ranging from 11 to 56%, highlighting the need for advancements in targeted and immunotherapy for this subtype [31]. Our analysis revealed high MZB1 expression across various ovarian cancer cell lines, particularly in OCCC, which contrasts with our understanding that MZB1 is predominantly distributed in immune cells [32,33]. Our pathway analysis showing MZB1’s involvement in HIPPO and WNT signaling provides a plausible mechanism, as these pathways are frequently dysregulated in OCCC. Notably, our GSEA links phenotypic changes to MZB1’s regulation of DNA damage response and cell cycle pathways, which aligns with recent findings in colorectal cancer, where MZB1 deficiency promotes genomic instability [15]. MZB1 is associated with DNA repair pathways [34,35]. Altered expression of MZB1 in tumor cells is associated with DNA damage response and cell cycle regulation, highlighting its multifaceted roles in ovarian cancer and suggesting its potential as a therapeutic target [36]. PARPi exhibits their effects against tumors with DNA repair deficiencies [37]. MZB1’s association with DNA damage repair response indicates its potential to predict PARPi efficacy [38]. Developing drugs targeting MZB1, in combination with PARPi, could improve treatment outcomes for OCCC [39]. Furthermore, combining immune checkpoint inhibitors with PARPi holds promise, and MZB1 may serve as a predictive marker for this combination therapy [40].

Despite these advances, our study has limitations. First, the overrepresentation of high-grade serous carcinoma in the TCGA cohort necessitates validation in larger cohorts. Second, the *in vitro* findings require confirmation through *in vivo* models and mechanistic studies to delineate MZB1’s precise roles in DNA repair versus immune modulation. Third, the clinical applicability of the proposed nomogram needs external validation across diverse populations.

## 5 Conclusion

In conclusion, MZB1 can inhibit tumor cell proliferation and migration, thereby impacting ovarian cancer prognosis and correlating with immune infiltration. Our study elucidates the multifaceted roles of MZB1 in ovarian cancer cells and the TIME, providing experimental evidence that supports its potential as a therapeutic target. We developed a prognostic model incorporating MZB1, significantly enhancing predictive accuracy for patient outcomes and providing clinicians with powerful tools to develop tailored treatment strategies. Moving forward, we anticipate that further research will clarify the specific mechanisms by which MZB1 influences ovarian cancer progression, ultimately advancing its clinical translation and application in ovarian cancer treatment.

## Supplementary Material

Supplementary Figure

Supplementary Table

## References

[1] Kuroki L, Guntupalli SR. Treatment of epithelial ovarian cancer. BMJ. 2020;371:m3773. 10.1136/bmj.m3773 33168565

[2] Gao J, Han W, He Y, Zhou J, Miao J, Zhang G. Livin promotes tumor progression through YAP activation in ovarian cancer. Am J Cancer Res. 2020;10(10):3179–93. PMC7642671 33163264

[3] Konstantinopoulos PA, Matulonis UA. Clinical and translational advances in ovarian cancer therapy. Nat Cancer. 2023;4(9):1239–57. 10.1038/s43018-023-00617-9 37653142

[4] Wang Z, Zhang W, Fang J, Xie P, Miao M, Yang H. Circular RNA circEXOC6B inhibits the progression of ovarian cancer by sponging miR-421 and regulating RUS1 expression. Onco Targets Ther. 2020;13:8233–43. 10.2147/OTT.S243040 PMC7443403 32884301

[5] Coleman RL, Monk BJ, Sood AK, Herzog TJ. Latest research and treatment of advanced-stage epithelial ovarian cancer. Nat Rev Clin Oncol. 2013;10(4):211–24. 10.1038/nrclinonc.2013.5 PMC3786558 23381004

[6] Del CJ, Matulonis UA, Malander S, Provencher D, Mahner S, Follana P, et al. Niraparib maintenance therapy in patients with recurrent ovarian cancer after a partial response to the last platinum-based chemotherapy in the ENGOT-OV16/NOVA trial. J Clin Oncol. 2019;37(32):2968–73. 10.1200/JCO.18.02238 PMC6839909 31173551

[7] Gonzalez-Martin A, Pothuri B, Vergote I, Graybill W, Lorusso D, McCormick CC, et al. Progression-free survival and safety at 3.5 years of follow-up: results from the randomised phase 3 PRIMA/ENGOT-OV26/GOG-3012 trial of niraparib maintenance treatment in patients with newly diagnosed ovarian cancer. Eur J Cancer. 2023;189:112908. 10.1016/j.ejca.2023.04.024 37263896

[8] Kim H, Xu H, George E, Hallberg D, Kumar S, Jagannathan V, et al. Combining PARP with ATR inhibition overcomes PARP inhibitor and platinum resistance in ovarian cancer models. Nat Commun. 2020;11(1):3726. 10.1038/s41467-020-17127-2 PMC7381609 32709856

[9] Wethington SL, Shah PD, Martin L, Tanyi JL, Latif N, Morgan M, et al. Combination ATR (ceralasertib) and PARP (olaparib) inhibitor (CAPRI) trial in acquired PARP inhibitor-resistant homologous recombination-deficient ovarian cancer. Clin Cancer Res. 2023;29(15):2800–7. 10.1158/1078-0432.CCR-22-2444 PMC11934101 37097611

[10] Lee EK, Matulonis UA. Emerging drugs for the treatment of ovarian cancer: A focused review of PARP inhibitors. Expert Opin Emerg Drugs. 2020;25(2):165–88. 10.1080/14728214.2020.1773791 32569489

[11] Peng Z, Li M, Li H, Gao Q. PD-1/PD-L1 immune checkpoint blockade in ovarian cancer: Dilemmas and opportunities. Drug Discov Today. 2023;28(8):103666. 10.1016/j.drudis.2023.103666 37302543

[12] Konstantinopoulos PA, Waggoner S, Vidal GA, Mita M, Moroney JW, Holloway R, et al. Single-arm phases 1 and 2 trial of niraparib in combination with pembrolizumab in patients with recurrent platinum-resistant ovarian carcinoma. JAMA Oncol. 2019;5(8):1141–9. 10.1001/jamaoncol.2019.1048 PMC6567832 31194228

[13] Wei H, Wang JY. Role of polymeric immunoglobulin receptor in IgA and IgM transcytosis. Int J Mol Sci. 2021;22(5):2284. 10.3390/ijms22052284 PMC7956327 33668983

[14] Suzuki K, Vogelzang A, Fagarasan S. MZB1 folding and unfolding the role of IgA. Proc Natl Acad Sci U S A. 2019;116(27):13163–5. 10.1073/pnas.1908012116 PMC6613165 31201222

[15] Wu W, Yang Z, Long F, Luo L, Deng Q, Wu J, et al. COL1A1 and MZB1 as the hub genes influenced the proliferation, invasion, migration and apoptosis of rectum adenocarcinoma cells by weighted correlation network analysis. Bioorg Chem. 2020;95:103457. 10.1016/j.bioorg.2019.103457 31901757

[16] Kanda M, Tanaka C, Kobayashi D, Tanaka H, Shimizu D, Shibata M, et al. Epigenetic suppression of the immunoregulator MZB1 is associated with the malignant phenotype of gastric cancer. Int J Cancer. 2016;139(10):2290–8. 10.1002/ijc.30286 27459504

[17] Yi C, Yang J, Zhang T, Qin L, Chen D. Identification of breast cancer subtypes based on endoplasmic reticulum stress-related genes and analysis of prognosis and immune microenvironment in breast cancer patients. Technol Cancer Res Treat. 2024;23:15330338241241484. 10.1177/15330338241241484 PMC11085026 38725284

[18] Chanukuppa V, Paul D, Taunk K, Chatterjee T, Sharma S, Shirolkar A, et al. Proteomics and functional study reveal marginal zone B and B1 cell specific protein as a candidate marker of multiple myeloma. Int J Oncol. 2020;57(1):325–37. 10.3892/ijo.2020.5056 32377723

[19] Tang Y, Feng X, Lu Q, Cui C, Yu M, Wen Z, et al. MZB1-mediated IgA secretion suppresses the development and progression of colorectal cancer triggered by gut inflammation. Mucosal Immunol. 2024;17(3):450–60. 10.1016/j.mucimm.2023.12.002 38101774

[20] Blanc-Durand F, Clemence WXL, Tan D. Targeting the immune microenvironment for ovarian cancer therapy. Front Immunol. 2023;14:1328651. 10.3389/fimmu.2023.1328651 PMC10757966 38164130

[21] Almeida-Nunes DL, Mendes-Frias A, Silvestre R, Dinis-Oliveira RJ, Ricardo S. Immune tumor microenvironment in ovarian cancer ascites. Int J Mol Sci. 2022;23(18):10692. 10.3390/ijms231810692 PMC9504085 36142615

[22] Yang C, Xia BR, Zhang ZC, Zhang YJ, Lou G, Jin WL. Immunotherapy for ovarian cancer: Adjuvant, combination, and neoadjuvant. Front Immunol. 2020;11:577869. 10.3389/fimmu.2020.577869 PMC7572849 33123161

[23] Jiang Y, Wang C, Zhou S. Targeting tumor microenvironment in ovarian cancer: Premise and promise. Biochim Biophys Acta Rev Cancer. 2020;1873(2):188361. 10.1016/j.bbcan.2020.188361 32234508

[24] Pitt JM, Marabelle A, Eggermont A, Soria JC, Kroemer G, Zitvogel L. Targeting the tumor microenvironment: Removing obstruction to anticancer immune responses and immunotherapy. Ann Oncol. 2016;27(8):1482–92. 10.1093/annonc/mdw168 27069014

[25] Xiao Y, Yu D. Tumor microenvironment as a therapeutic target in cancer. Pharmacol Ther. 2021;221:107753. 10.1016/j.pharmthera.2020.107753 PMC8084948 33259885

[26] Li D, Zhu Y, Zhang L, Shi L, Deng L, Ding Z, et al. MZB1 targeted by miR-185-5p inhibits the migration of human periodontal ligament cells through NF-kappaB signaling and promotes alveolar bone loss. J Periodontal Res. 2022;57(4):811–23. 10.1111/jre.13014 35653494

[27] Yang L, Xiong J, Li S, Liu X, Deng W, Liu W, et al. Mitochondrial metabolic reprogramming-mediated immunogenic cell death reveals immune and prognostic features of clear cell renal cell carcinoma. Front Oncol. 2023;13:1146657. 10.3389/fonc.2023.1146657 PMC10196130 37213288

[28] Gu S, Qian L, Zhang Y, Chen K, Li Y, Wang J, et al. Significance of intratumoral infiltration of B cells in cancer immunotherapy: From a single cell perspective. Biochim Biophys Acta Rev Cancer. 2021;1876(2):188632. 10.1016/j.bbcan.2021.188632 34626740

[29] Xu H, Li W, Zhu C, Cheng N, Li X, Hao F, et al. Proteomic profiling identifies novel diagnostic biomarkers and molecular subtypes for mucinous tubular and spindle cell carcinoma of the kidney. J Pathol. 2022;257(1):53–67. 10.1002/path.5869 PMC9311136 35043389

[30] Sun Y, Liu G. Endometriosis-associated Ovarian Clear Cell Carcinoma: A Special Entity? J Cancer. 2021;12(22):6773–86. 10.7150/jca.61107 PMC8518018 34659566

[31] Khalique S, Lord CJ, Banerjee S, Natrajan R. Translational genomics of ovarian clear cell carcinoma. Semin Cancer Biol. 2020;61:121–31. 10.1016/j.semcancer.2019.10.025 31698086

[32] Flach H, Rosenbaum M, Duchniewicz M, Kim S, Zhang SL, Cahalan MD, et al. Mzb1 protein regulates calcium homeostasis, antibody secretion, and integrin activation in innate-like B cells. Immunity. 2010;33(5):723–35. 10.1016/j.immuni.2010.11.013 PMC3125521 21093319

[33] Kapoor T, Corrado M, Pearce EL, Pearce EJ, Grosschedl R. MZB1 enables efficient interferon alpha secretion in stimulated plasmacytoid dendritic cells. Sci Rep. 2020;10(1):21626. 10.1038/s41598-020-78293-3 PMC7736851 33318509

[34] Xie Y, Kong W, Luo D, Chen S, Zhao X, Zhang HE. Ovarian clear cell carcinoma: Genomic characterization, pathogenesis and targeted therapy. Anticancer Res. 2023;43(8):3401–10. 10.21873/anticanres.16515 37500149

[35] Wong O, Li J, Cheung A. Targeting DNA damage response pathway in ovarian clear cell carcinoma. Front Oncol. 2021;11:666815. 10.3389/fonc.2021.666815 PMC8560708 34737943

[36] Mirza-Aghazadeh-Attari M, Ostadian C, Saei AA, Mihanfar A, Darband SG, Sadighparvar S, et al. DNA damage response and repair in ovarian cancer: Potential targets for therapeutic strategies. DNA Repair (Amst). 2019;80:59–84. 10.1016/j.dnarep.2019.06.005 31279973

[37] Washington CR, Moore KN. Resistance to poly (ADP-ribose) polymerase inhibitors (PARPi): Mechanisms and potential to reverse. Curr Oncol Rep. 2022;24(12):1685–93. 10.1007/s11912-022-01337-6 36346509

[38] Jurkovicova D, Neophytou CM, Gasparovic AC, Goncalves AC. DNA damage response in cancer therapy and resistance: Challenges and opportunities. Int J Mol Sci. 2022;23(23):2284. 10.3390/ijms232314672 PMC9735783 36499000

[39] O’Malley DM, Krivak TC, Kabil N, Munley J, Moore KN. PARP inhibitors in ovarian cancer: A review. Target Oncol. 2023;18(4):471–503. 10.1007/s11523-023-00970-w PMC10344972 37268756

[40] Wu Z, Cui P, Tao H, Zhang S, Ma J, Liu Z, et al. The synergistic effect of PARP inhibitors and immune checkpoint inhibitors. Clin Med Insights Oncol. 2021;15:1179554921996288. 10.1177/1179554921996288 PMC7934064 33737855

