# Supplementary material for: MZB1 regulates the immune microenvironment and inhibits ovarian cancer cell migration
Source: Open Med (Wars). 2025 May 13;20(1):20251174. doi: 10.1515/med-2025-1174 (PMC12086629; doi:10.1515/med-2025-1174)
Supplement: Supplementary Figure [file med-2025-1174-sm.pdf]

Supplementary material

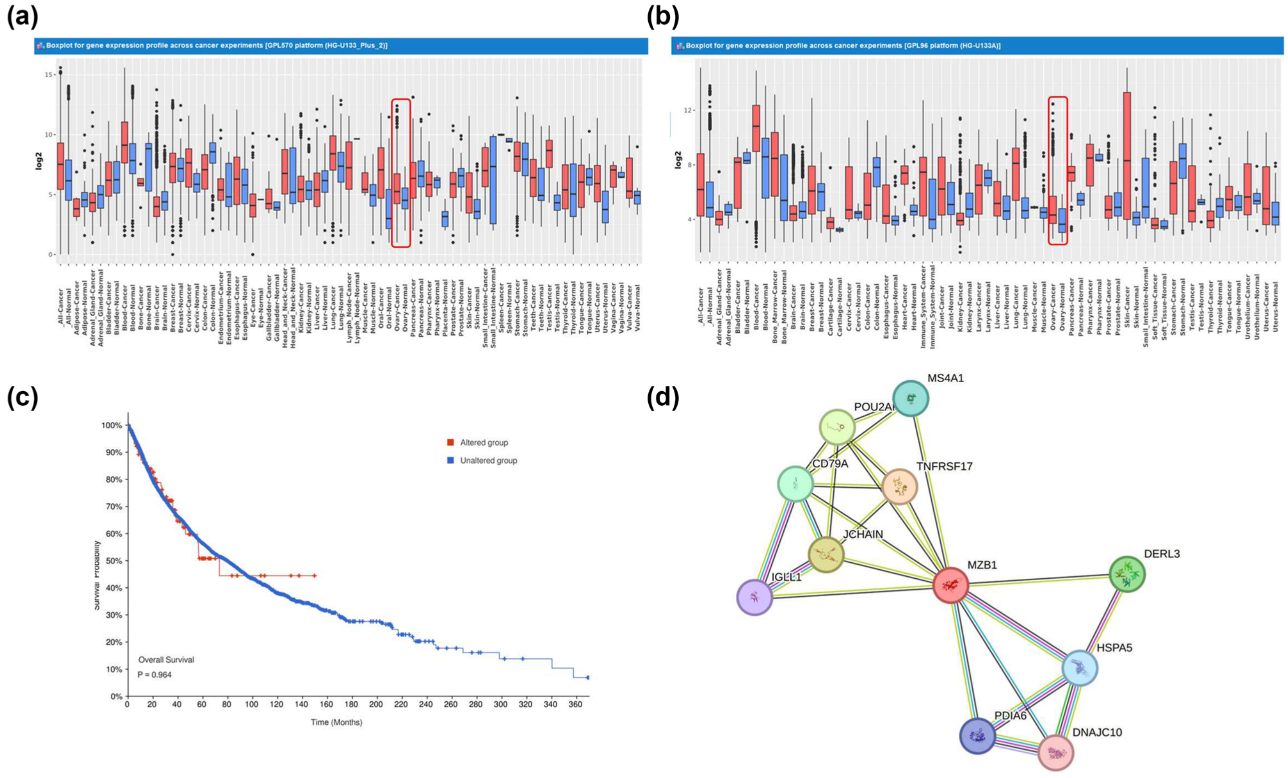

Figure S1: (a) The expression profile of MZB1 in various tumors in GEO [GPL570 platform (HG-U133 PIus 2]; (b) the expression profile of MZB1 in various tumors in GEO [GPL96 platform (HG-U133A)]; (c) potential correlation between mutation status and overall survival; (d) the detailed protein interaction of MZB1 was revealed by the PPI network.

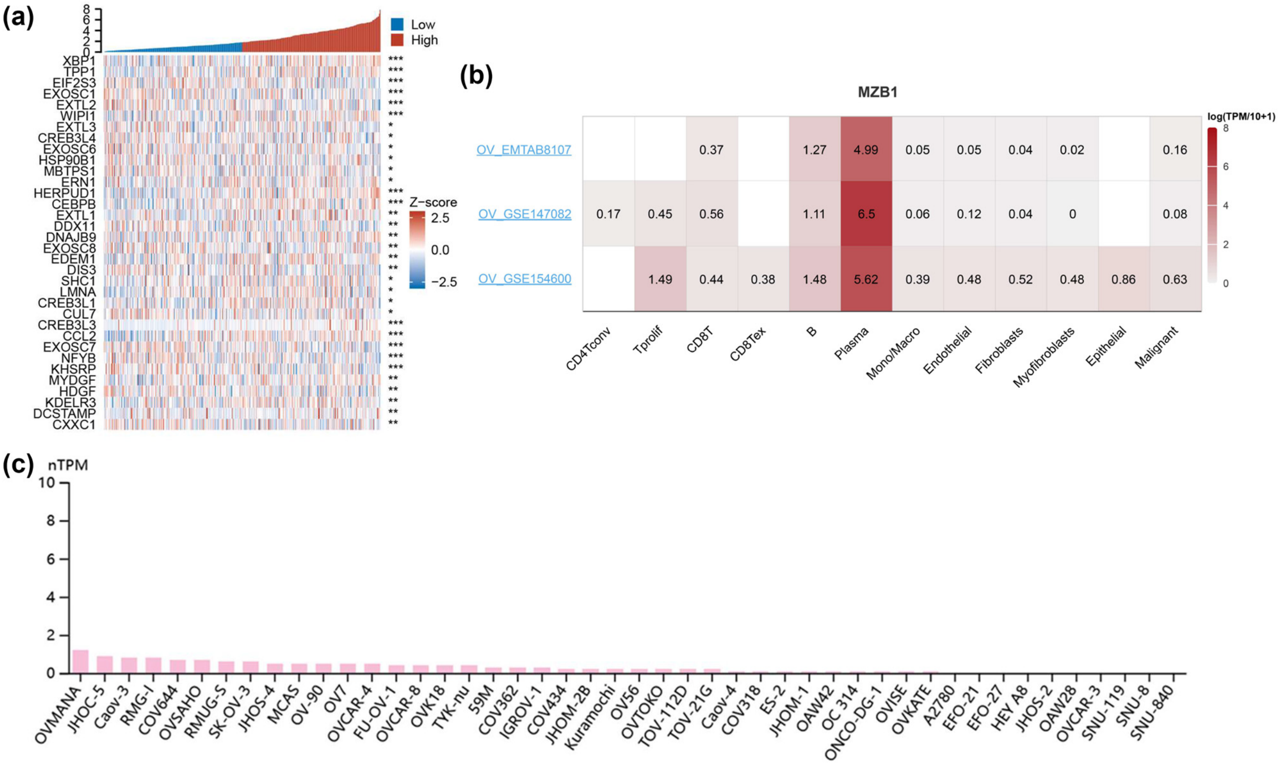

Figure S2: (a) Differences in the expression of endoplasmic reticulum stress-related proteins in MZB1 high-expression and low-expression groups; (b) expression of MZB1 in individual cells of ovarian cancer single-cell database; (c) expression of MZB1 in multiple ovarian cancer cell lines. **P* < 0.05; ***P* < 0.01; ****P* < 0.001.

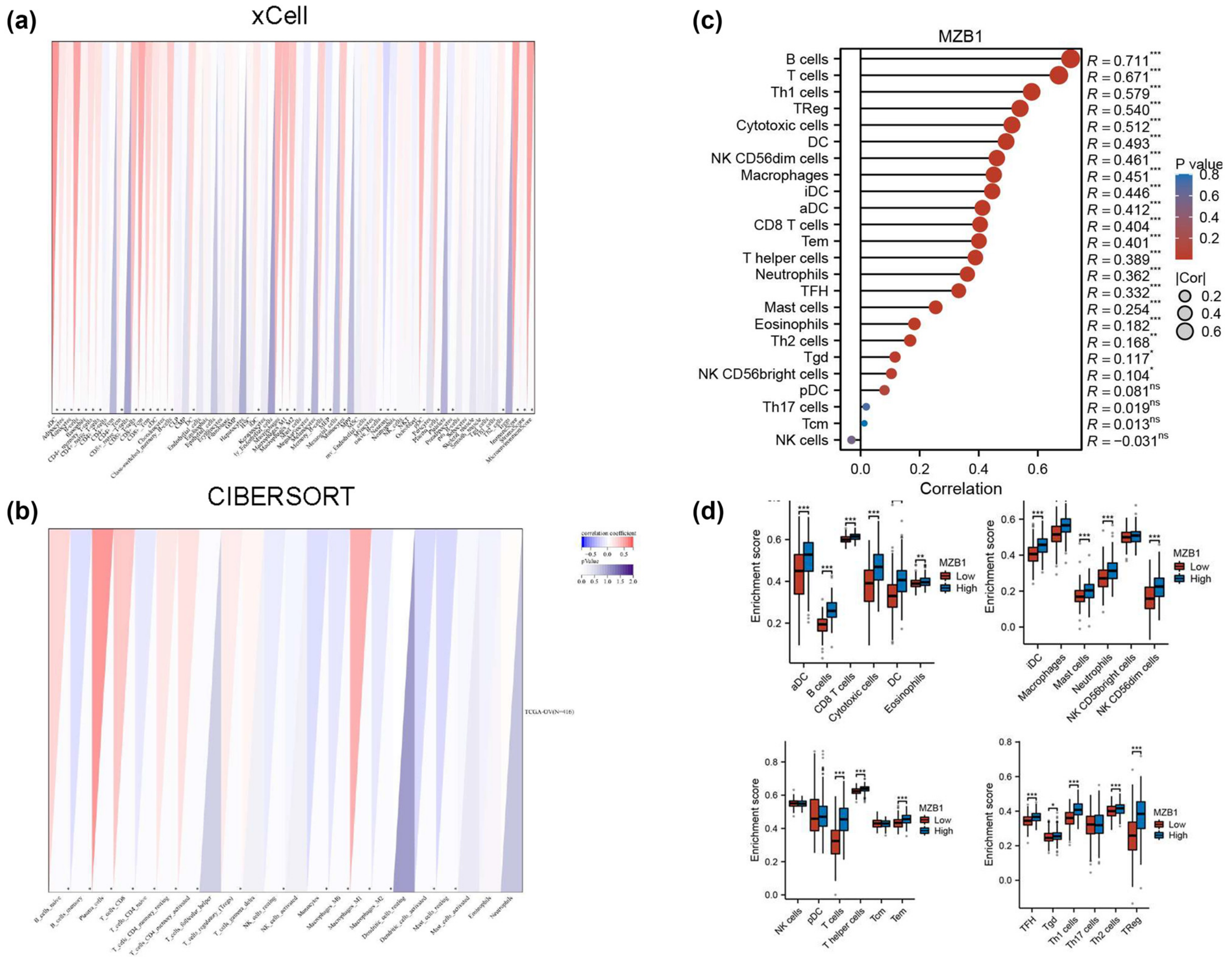

Figure S3: MZB1 is associated with multiple immune cells. (a) and (b). Xcell and CIBERSORT algorithms were used to perform immune cell infiltration analysis; (c) and (d). The correlation between MZB1 and various immune cells. *Spearman*; $*P < 0.05$; $**P < 0.01$; $***P < 0.001$.

(a) TargetScan

	Predicted consequential pairing of target region (top) and miRNA (bottom)	Site type	Context++ score	Context++ score percentile	Weighted context++ score	Conserved branch length	P_{CT}	Predicted relative K_D
Position 189-195 of MZB1 3' UTR<br>hsa-miR-370-5p	5' ...CUCAAAUAAAACCCAGUGACCUC...<br>||||||| 3' CAUUGACGUCUCUGCACUGGAC	7mer-m8	-0.41	99	-0.41	3.154	N/A	-5.275
Position 189-195 of MZB1 3' UTR<br>hsa-miR-1193	5' ...CUCAAAUAAAACCCAGUGACCUC...<br>|||| ||||||| 3' CCUAUCAGUUUGCC-CACUGGAU	7mer-m8	-0.47	99	-0.47	3.154	N/A	-4.351

(b) mirDIP

Links	Gene Symbol	Uniprot	Pseudogene	microRNA	Integrated Score	Number of Sources	Score Class
GC UP	MZB1	Q8WU39		hsa-miR-1193	0.512147443565461	6	Very High
GC UP	MZB1	Q8WU39		Hsa-Mir-3909_5p*	0.123881709860319	5	High

Figure S4: miR-1193 regulates the expression of MZB1. (a) TargetScan web tool to predict potential target genes; (b) mirDIP web tool to predict potential target genes.
